# Supplementary material for: The pioneering gut microbiome acquired via different delivery modes in neonates shapes distinct immune and metabolic environments
Source: medRxiv. 2025 Oct 2:2025.09.30.25337005. Preprint. [Version 1] doi: 10.1101/2025.09.30.25337005 (PMC12747249; doi:10.1101/2025.09.30.25337005)
Supplement: Supplement 1 [file NIHPP2025.09.30.25337005v1-supplement-1.pdf]

medRxiv preprint doi: https://doi.org/10.1101/2025.09.30.25337005; this version posted October 2, 2025. The copyright holder for this preprint (which was not certified by peer review) is the author/funder, who has granted medRxiv a license to display the preprint in perpetuity.
This article is a US Government work. It is not subject to copyright under 17 USC 105 and is also made available for use under a CC0 license.

Supplemental Table 1: Infant and maternal clinical characteristics of human subjects

	**Vaginally delivered (VD)** (n=4)	**Cesarean section delivered (CS)** (n=3)	**Cesarean delivered and vaginally seeded (CS+VS)** (n=3)
**Infant Characteristics**			
Gestational age in weeks (mean)	38.95	39	38.67
Breast milk prior to stool sample, n (%)	4 (100%)	3 (100%)	3 (100%)
Formula prior to stool sample, n (%)	2 (50%)	0 (0%)	1 (33%)
Antibiotics prior to stool sample, n (%)	0 (0%)	0 (0%)	0 (0%)
Sex female, n (%)	2 (50%)	2 (67%)	0 (0%)
**Maternal Characteristics, n (%)**			
Pre-pregnancy body mass index			
< 25	2 (50%)	3 (100%)	1 (33%)
25-30	2 (50%)	0 (0%)	1 (33%)
>30	0 (0%)	0 (0%)	1 (33%)
Prenatal Antibiotics	0 (0%)	0 (0%)	0 (0%)
Peripartum Antibiotics	0 (0%)	3 (100%)	3 (100%)
Race			
White	3 (75%)	3 (100%)	1 (33%)
Black or African American	0 (0%)	0 (0%)	1 (33%)
More than one race/Other/Decline to answer	1 (25%)	0 (0%)	1 (33%)
Ethnicity			
Hispanic or Latino	1(25%)	0 (0%)	1 (33%)
Not Hispanic or Latino/Other	3 (75%)	3 (100%)	2 (67%)
